# Artificial Warming Facilitates Growth but Not Survival of Plateau Frog (*Rana kukunoris*) Tadpoles in Presence of Gape-Limited Predatory Beetles

**DOI:** 10.1371/journal.pone.0098252

**Published:** 2014-06-06

**Authors:** Jiyan Zhao, Yangheshan Yang, Xinqiang Xi, Changbing Zhang, Shucun Sun

**Affiliations:** 1 Department of Biology, Nanjing University, Nanjing, China; 2 ECORES Lab, Chengdu Institute of Biology, Chinese Academy of Sciences, Chengdu, China; 3 Sichuan Academy of Grassland Science, Chengdu, China; Tennessee State University, United States of America

## Abstract

**Background:**

Global warming has been frequently demonstrated to increase growth rate in larval amphibians that have considerable phenotypic plasticity; this may lead to an increase in larval survival because large larvae are less likely to be captured by gape-limited predators. This study is to test whether warming could improve tadpole growth and thereby enhance the tadpole survival in plateau frog *Rana kukunoris*.

**Methodology:**

We conducted an experiment involving growing tadpoles under two contrasting temperatures, i.e. ambient temperature vs. warming by 3.8°C, with and without their major predators – the gape-limited predaceous diving beetles *Agabus* sp. in eastern Tibetan Plateau, in a factorial arrangement. We recorded the survival and measured body fresh weight and morphological characteristics of the tadpoles.

**Principal Findings:**

Warming significantly increased body fresh weight in the presence of predators after three weeks of treatments. However, the predators imposed significant and similar effects on the survival of tadpoles under both ambient and elevated temperatures, with the effects mostly occurring in the first three weeks of the experiment. Changes in the body form, i.e. the greater whole length at a given fresh weight and the longer tail at a given body length, could have acted as mechanisms of defense and escape for the tadpoles.

**Conclusions/Significance:**

Warming did not increase tadpole survival with or without presence of predators. Moreover, an increased growth rate (due to warming in the presence of predators) was not a major factor contributing to the tadpole survival. We postulate that even if warming increases the tadpole growth rate in the plateau frog, it does not necessarily improve their survival in the presence of gape-limited predators.

## Introduction

Global average surface temperature had increased by 0.85°C from 1880 to 2012 [1] and is predicted to further rise by 2 to 6°C at the end of the 21st century [2]. Global warming may significantly change the behavior, growth rate, and reproductive phenology of a species, and further affect its relationship with other interacting species. In particular, ectothermic organisms are sensitive to temperature due to lack of an efficient mechanism for physiological thermoregulation [3]; therefore interactions (e.g. competition and predation) among these species are apt to vary with temperature [4], [5]. Although the declines and even extinction of some amphibian species world-wide are frequently attributed to climate change in recent decades [6], few studies have explicitly addressed the warming effect on predation-prey interactions involving amphibians and other ectothermic animals (but see [5]).

Amphibians are well known to have complex life cycles and often exhibit high phenotypic plasticity [7], [8], depending on the physical (e.g. temperature, rainfall, and water depth) and biological conditions (e.g. density, predator [9]) of environments. Larval amphibians – tadpoles – often suffer the highest mortality during life history [10], [11]. Numerous studies have suggested predation as a major source of the mortality in larval populations of both anurans and urodeles [7], [11]. Tadpoles may employ various defensive mechanisms against predators, including growing long tails to increase swimming ability and strong muscles to physically fend off predators [12], [13], advancing the timing of metamorphosis to leave aquatic habitats [7], and increasing growth rate to become too large for predators [5], [14], [15]. Similarly, predators have also evolved effective techniques (e.g. physical visions and chemical signals) to detect, capture and handling the defensive prey [14]. Theoretically, if warming causes a slight change in the defensive traits or activities in either one of the players, the tadpole-predator interaction and associated tadpole survival may be significantly altered.

Several lines of evidence have suggested that temperature increase may largely increase tadpole growth rate in many amphibian species. For example, long-term field monitoring programs [16], [17] have revealed that tadpoles grow faster in warm years than in cold years; large spatial-scale investigations have shown that the growth rate of tadpoles progressively increases across the gradients from high to low latitudes or altitudes [18]–[20]; laboratory studies have also indicated that amphibian tadpoles grow faster at high temperatures [5], [21], [22]. Another issue is that many predator species preying on tadpoles are gape-limited killers such as diving beetles (e.g. *Acilicus sulcatus*) and dragonfly larvae (e.g. *Pantala flavescens*) in ephemeral ponds, which are among the fiercest predators in aquatic environment. For these predators, their ability of feeding on larval amphibians often decreases with increasing tadpole size [23] simply because their gapes are not large enough to capture big tadpoles [10], [24], [25]. Thus, Anderson et al. [5] reasonably hypothesized that warming should increase tadpole survival in the presence of these gape-limited predators.

This “warming–fast growth–high survival” hypothesis has been supported by direct and indirect evidence. For example, field investigations suggest that both growth rate and tadpole survival are positively correlated with temperature across years [19], and laboratory experiments show similar findings that increased temperature often facilitates both tadpole growth and survival [21], [26]. However, this hypothesis may suffer possible flaws because it largely overlooks the response of other defensive traits to warming. For example, warming may change the body form of tadpoles (e.g. the ratio of tail length to body length and the ratio of tail muscle depth to tail length), which may further affect their swimming performance. Tadpoles with larger and deeper tail fins relative to body size generally have greater burst speed and maneuverability [12], [27] and more likely to evade predators [12], [28], [29]. Few empirical experiments have simultaneously examined tadpole growth and other defensive traits under warming in the presence of predators, such that this “warming–fast growth–high survival” hypothesis needs to be further tested as whether increased growth rate truly significantly contributes to tadpole survival without involving alterations of other defensive traits.

Warming in alpine regions is predicted to be stronger than in other places and Tibetan plateau has experienced the most significant warming over the past 50 years [30], [31]. There are extensive alpine meadows in the eastern part on the plateau [32]. *Rana kukunoris*, a plateau frog, is the most common top-predator species in alpine meadows of Zoige regions in eastern Tibet Plateau. The tadpoles of this frog are fragile and vulnerable to predators, as shown in several recent studies [33], [34]. In this study, we conducted a factorial experiment (2 factors x 2 levels) involving growing the tadpoles of the plateau frog in presence or absence of the predators (diving beetle) interacting with ambient temperature and warming treatments. The predatory beetle species is gape size-limited and could not directly capture large tadpoles. We examined the survival, growth rate, and body form of the tadpoles at different growth phases during the experiment. We asked the question whether warming could increase the growth and survival rate of the tadpoles in the very early life stage and, if so, whether increased growth rate would contribute to increased survival.

## Methods and Materials

### Ethics Statement

Our research on the plateau brown frog *Rana kukunoris* and diving beetles (*Agabus* sp., Coleoptera: Dytiscidae) in the alpine meadow was approved by the Chinese Wildlife Management Authority and conducted under Law of the People's Republic of China on the Protection of Wildlife (August 28, 2004). No permits were required to carry out this study. All animal work was approved by the Animal Care Committee at Nanjing University.

### Study site and species

Our study site is situated in Hongyuan County of Sichuan Province, Southwest China (32°48′N, 102°33′E). The climate is of cold and continental type, and is characterized by short and cool spring, summer, and autumn, and a long winter. During the period 1961–2008, according to data from the Meteorological Station of Hongyuan County, which is located 5 km from the study site, the annual mean temperature was 0.9°C, with maximum and minimum monthly means of 10.9°C and −10.3°C in July and January, respectively. The annual mean precipitation was 690 mm (80% of which occurs between May and August).

We selected tadpoles of *Rana kukunoris* as prey and the predaceous diving beetles (*Agabus* sp., Coleoptera: Dytiscidae) as predator species. The frog species is endemic to China, living on alpine meadows that are interspersed with ponds or marshes at altitudes ranging 3000–4000 m on the eastern part of the Tibetan Plateau. The population density and distribution area of *Rana kukunoris* are declining presumably due to human activities and global warming [33], [34]. The adults of *Rana kukunoris* are 4–6 cm in length, with females being often larger than males, and spawn from mid-April to early July, lasting for about three months; they lay eggs in still water or in the puddle of brooks. They often overwinter in the local puddles [35]. The larvae and tadpoles are small and brown. The food spectrum of tadpoles includes algae, mosquito larvae, and water flea; sometimes tadpoles are observed to consume the corpse of earthworms when food is not sufficient. The natural enemy of tadpoles include dragonfly larvae, diving beetle (both adults and larvae), and boatmen (*Corixa* sp.).

The diving beetle is 0.8–1.5 cm in length for adults and about 2–5 cm in length for larvae (also called water tigers). The females usually lay eggs within stalks of aquatic plants and the eggs pupate in moist soils. Both adults and larvae are fierce predators to the tadpoles of *Rana kukunoris* [36]: the adults usually attack small tadpoles, presenting as a gape-limited predator, while the larvae seem to be non-selective on tadpole size. The beetle density varies with season, pond size and water depth. An independent field survey, which was conducted in May 2013, showed that the density ratio of tadpoles to beetles ranged from 50 to 500 during the periods of frog spawning and tadpole growth.

### The warming experiment

The experiment was in factorial arrangement involving two factors at two levels each (i.e. predator presence vs. absence, and ambient temperature vs. warming), resulting in four treatments, i.e. warming with presence of predator (W+P+), ambient temperature with presence of predator (W−P+), warming without predator (W+P−), and ambient temperature without predator (W−P−). Our major objective was to determine the warming effect on predator-tadpole interactions, which can be deduced from the difference between W−P+ and W+P+ treatments. The W−P− and W+P-treatments served as a complementary control to allow for determining whether nature mortality significantly contributed to tadpole survival in the presence of predators and to test whether warming affected tadpole growth if the tadpole density was not significantly changed by warming. Each treatment was replicated five times, and each replicate contained fifty tadpoles hatched from eggs two days earlier, with the initial average size being ca. 9.2 mm in length and ca. 1.0 mg in fresh mass (Appendix S2). One adult diving beetle, the size of ca. 15 mm, was placed within each replicate of treatments with predator. The ratio of tadpoles to beetle larvae is close to the upper end of the ratio range; this is assumed to include the predation risks from other predators, but not too high for ensuring that tadpoles would not eaten up by the diving beetles in a very short time.

We collected egg masses of the plateau frog from twenty ponds scattered on the meadows in the study area; the eggs were laid one day prior to the collection and the quantity of eggs collected was large enough to meet the experimental requirement. Each egg mass was put into one bucket (15 cm in bottom diameter and 15 cm tall) filled with water collected from a well for supplying drinking water for humans and animals, and then placed outdoor for natural hatching. When more than 80% of the eggs were hatched, two or three emerged tadpoles from each egg mass were placed into tanks (experimental replicates). Meanwhile, twenty diving beetles were collected from the same ponds for collection of frog eggs. After being reared with tadpoles for two days, ten adult beetles were selected and placed into 10 tanks that were used for predator-present treatments. The experimental tanks were made of plastic and transparent, each with a dimension of 33 cm×23 cm×19 cm (high). Each tank contained 7 L clean groundwater collected from the well, which had been placed in large chambers for >24 hrs. The water was replaced weekly. Tadpoles were fed with a finely ground fish food comprised of mixture of bean cake and wheat bran (3∶1). The food rations increased from 25 to 70 mg per tadpole per day as the experiment progressed; this high ratio ensured food availability being non-limiting. All tanks were added with 20 g pond vegetation (*Potamogeton wright)* as perching sites for the predator and tadpoles. The plants kept green and showed no sign of decay during the experiment. All the survived tadpoles and diving beetles were released to the field after the experiment was terminated. No tadpoles metamorphosed until the end of the experiment; the duration of tadpole stage was about 50 days even under the warming treatments.

Artificial warming was achieved by placing half (N = 10) of the tanks into a greenhouse, with the remaining half (N = 10) placed outdoor under an overtop glass shelter. Measurements with temperature sensors (JL-04 logger, Handan, China) showed that the mean temperature in the glasshouse was 16.5°C during the experiment, which was 3.8°C higher than the mean outdoor temperature (see Appendix S1 for details). The magnitude of the temperature increase is more than half of the predicted temperature increase (≈6°C) by IPCC [2] for the Tibetan Plateau at the end of this century.

### Response variables

After commencement of the experiment, we recorded the number of surviving tadpoles in each tank and measured the body length and tail length of each tadpole following the method of Benard [29]; the fresh weight of the surviving tadpoles was also determined. The whole length was calculated as the sum of body length and tail length. These investigations were conducted 12 hrs after commencement of the experiment (on May 11, 2013), and thereafter at weekly intervals until the fourth week, as there was no further change in the survival of tadpoles in the fifth week. Tadpole survival was additionally recorded 24 hrs and 48 hrs after the experiment started assuming that tadpole mortality was high in the early stage of development.

At each time of measurements, all the tadpoles from each tank were put into a glass petri dish with a little water so that the body naturally stretched, and then they were individually photographed using a Samsung digital camera (SamsungPL620, Samsung Electronics Co LTD, Korean) while a ruler was placed besides as scale for calibration. All photographs were taken with the same shutter, aperture, focal distant, and exposure compensation through manual control, following Touchon and Warkentin [37]. Subsequently, all the tadpoles were weighed in the entirety for each tank after removing the water on the body surface of the tadpoles. The measurement procedure typically took less than 2 minutes, which was assumed to have no significant negative impact on growth and development of tadpoles [29]. All the photographs were analyzed using ImageJ to obtain the morphological parameters.

There was no mortality in all the diving beetles during the experiment. The growth of the beetles during the experiment was non-significant in terms of both body fresh weight (F_(1,18)_ = 0.10, P = 0.754) and body length (F_(1,18)_ = 0.11, P = 0.744).

### Data analysis

The measured variables were first averaged for each tank and then for each treatment. All the data of interest were tested for normality and variance heterogeneity before analyses. Tadpole fresh weight was log-transformed to achieve normality. The effects of predator and warming, as well as their interaction, on tadpole survival, growth and morphology were determined using two-way ANOVAs by observation time. If significant effects were detected, *post hoc* Tukey tests were used to determine the difference between treatments. All the statistical analyses were carried out in R program [38].

In order to test whether predator had induced defensive traits, we determined the scaling relationships between tadpole body length and tail length using Standardized Major Axes (SMA) line fitting because there was a possibility of measurement error in both x and y variables and allometric slopes were of particular interest. Slopes were calculated as SMA [39]; differences in the elevation of regression slopes (y-intercept) and in shifting along the common slope were tested by ANOVA (and *post hoc* Tukey tests where appropriate). The scaling relationships were determined based on the pooled data collected in three days after the beginning of the experiment, during which predator-induced defensive traits were present (see results). Computation of the allometric equation parameters was conducted using (S)MATR Version 2.0 [40].

## Results

### Tadpole survival and growth

**Figure 1:**
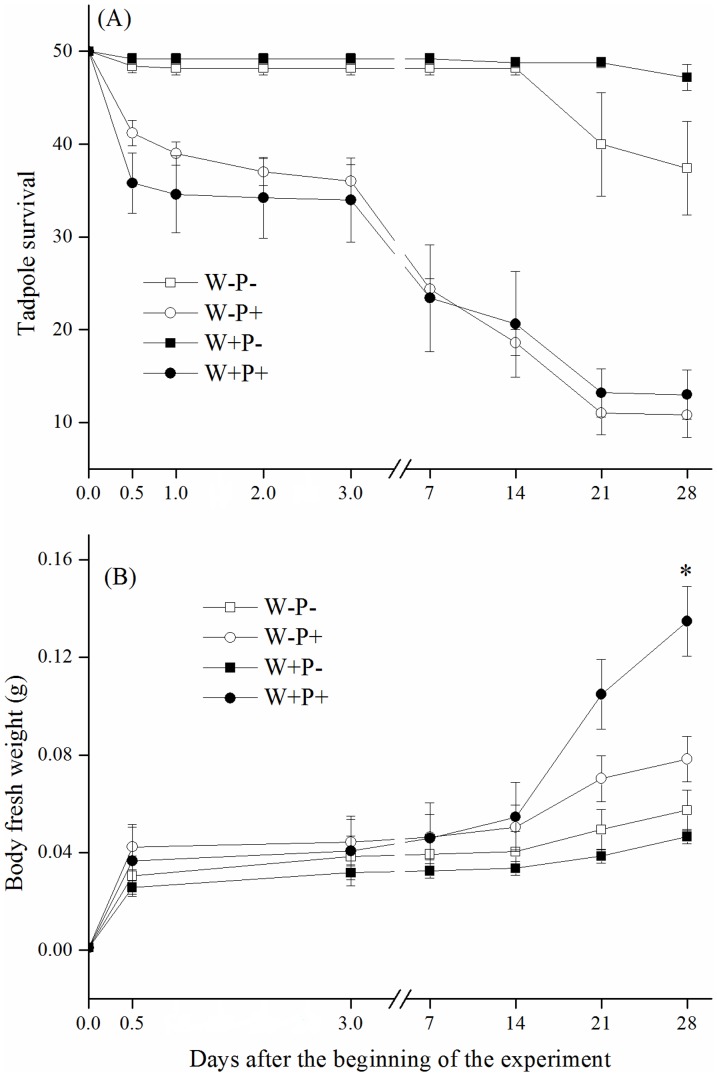
Tadpole survival (A) and body fresh weight (B) in four treatments including warmed and predator present (W+P+), ambient and predator present (W−P+), warmed and predator absent (W+P−), and ambient and predator absent (W−P−). The asterisk denotes a statistically significant difference between warmed and ambient treatments at the 0.05 level, as derived from a two-way ANOVA followed by *post hoc* Tukey's test on the observation day. Data are means ±1SE.

Predator significantly decreased the tadpole survival in both warming and ambient temperature treatments (Appendix S3), particularly in the first three weeks, during which more than 90% of the reduction in the survival occurred (Fig. 1A); thereafter the survival kept constant presumably due to the gape-limitation in the predators. However, the warming effect was generally non-significant on tadpole survival, and no significant difference was observed between the two predator-absence treatments and between the two predator-presence treatments over the duration of the experiment (Fig. 1A, Appendix S3). Notably, in the absence of predators the tadpole survival was high and did not differ between the warming and ambient temperature treatments, suggesting that natural mortality was not a significant contributor to the variation in tadpole survival in the predator-presence treatments.

No significant difference was found in the tadpole fresh weight between the warming and ambient temperature treatments (*P*>0.05 for each observation) until the fourth week after commencement of the experiment (Fig. 1B). On the final observation, the fresh weight of tadpoles differed significantly between the ambient temperature and warming treatments on in the presence of predators (Fig. 1B; Appendix S3).

### Tadpole morphology

**Figure 2:**
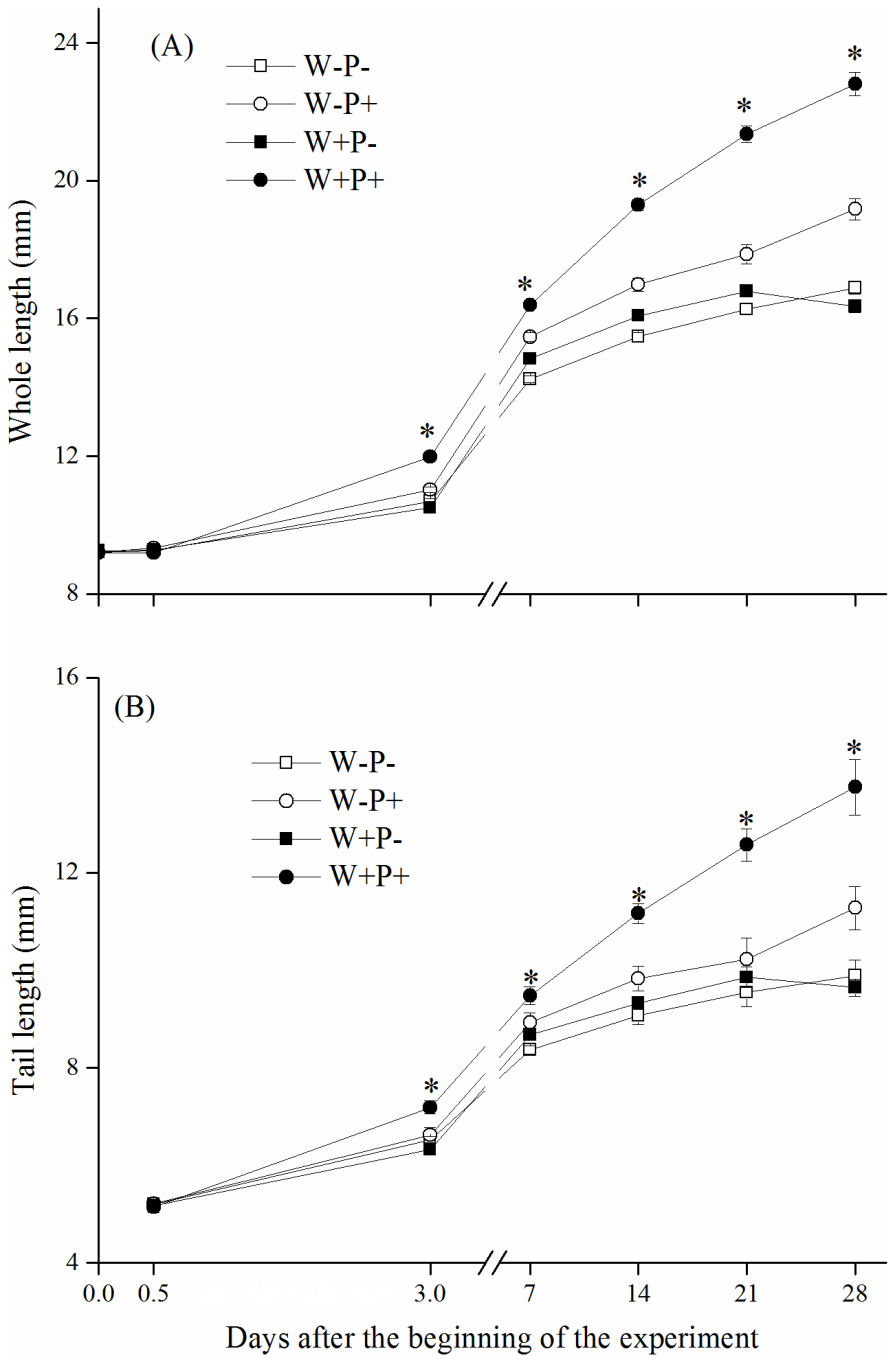
Whole length (A) and tail length (B) of tadpoles in four treatments including warmed and predator present (W+P+), ambient and predator present (W−P+), warmed and predator absent (W+P−), and ambient and predator absent (W−P−). The asterisks denote statistically significant differences between warmed and ambient treatments at the 0.05 level, as derived from a two-way ANOVA followed by *post hoc* Tukey's tests on observation days. Data are means ±1SE.

The whole length of individual tadpoles responded to the experimental factors earlier than body growth rate (reflected by body fresh weight). Starting from the third day of the experiment, the whole length of individual tadpoles was significantly longer in the warming treatment than in the ambient temperature treatment in the presence of predator, but not in the treatments without predator (Fig. 2A, Appendix S3). A similar tendency was also observed in the tadpole tail length (Fig. 2B, Appendix S3).

**Figure 3:**
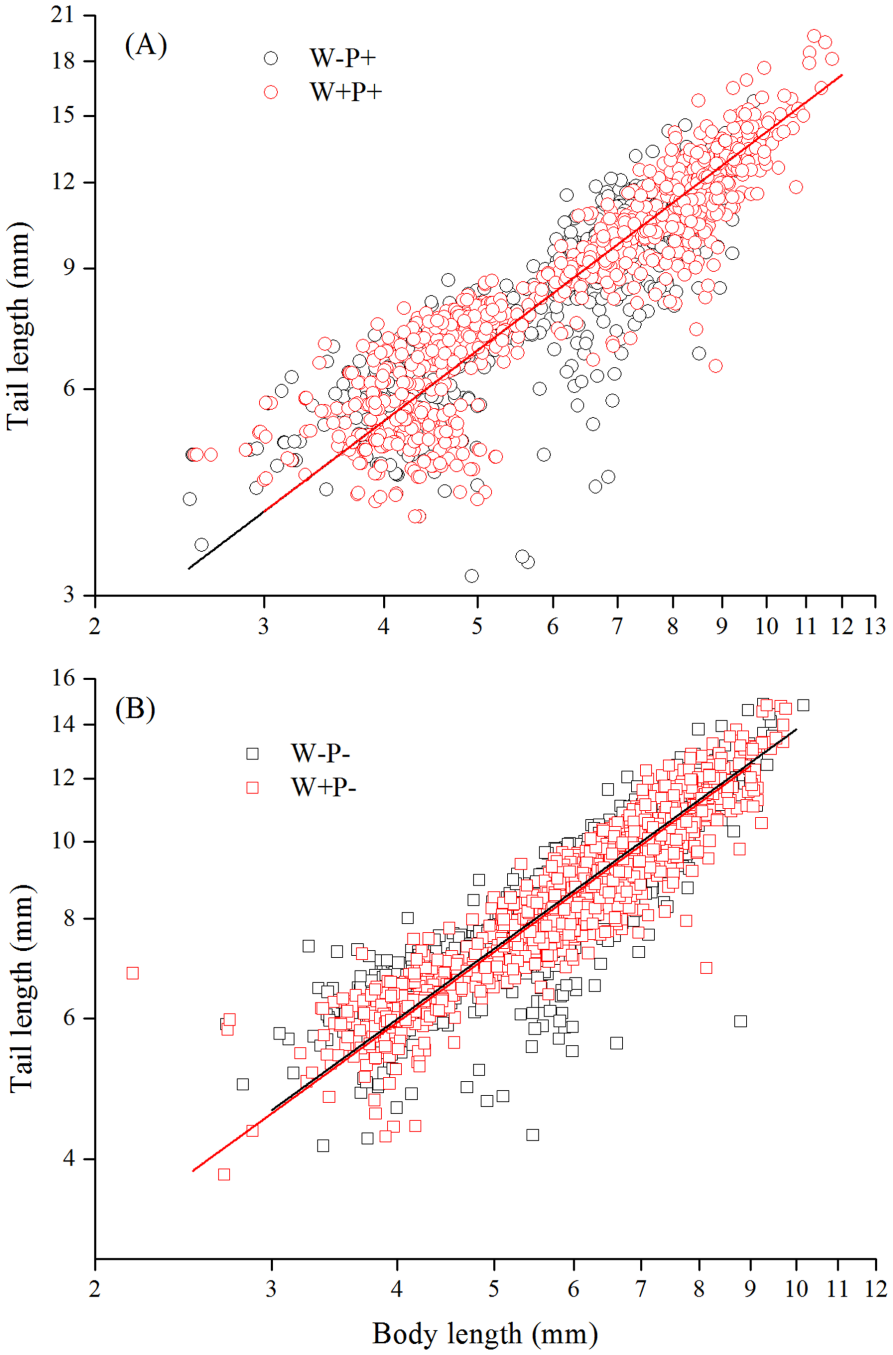
The scaling relationships between tail length and body length (A and B) in the presence and absence of predators. In (A) the warmed and ambient treatments share common slope and y-intercept, but the warmed has a significant shift along the common slope. In (B), the warmed and ambient treatments share a common slope, but y-intercept is significantly higher in the warmed than the ambient. See Appendix S4 for the equation fitting and see text for the difference between treatments. W+P+, warmed and predator present; W−P+, ambient and predator present; W+P−, warmed and predator absent; W−P−, ambient and predator absent.

Body form was also significantly modified by warming. In the presence of predator, tadpoles of the warming and ambient temperature treatments shared a common slope (not significantly different from 1.0) in the scaling relationships between body length and tail length (Appendix S4), but the tadpoles in the warming treatment displayed an upright shift along the common slope (i.e. shift in both X and Y axis; Fig. 3A), suggesting that at a given body length, the tail length was indistinguishable between the warming and ambient temperature treatments. However, in the absence of predators, the y-intercept of the common slope in the scaling relationships between body length and tail length was significantly higher in the ambient temperature treatment than in the warming treatment (Fig. 3B), suggesting that at a given body length, the tail length was greater under ambient temperature than in a warming environment.

## Discussion

### Tadpole growth

Warming did not impose significant effect on tadpole growth in predator-free environment in this study, inconsistent with a widely accepted dogma that warming facilitates tadpole growth. High temperature is often assumed to favor tadpole growth as in other ectotherms [26], [41], [42]. Nevertheless, our study is not the only case violating this dogma. Touchon and Warkentin [22] also found that the single warming effect on tadpole growth was not significant in Neotropical treefrog *Dendropsophus ebraccatus* over a three-week long experiment. Moreover, Alvarez and Nicieza [21] reported that tadpole body mass of the Iberian Painted Frogs (*Discoglossus galganoi*) varied inversely with temperature under non-limiting food availability. In the present study, food availability was not limiting so that the tadpoles reared under ambient temperature were able to obtain sufficient energy to maintain their metabolism and growth. We observed that tadpoles in the warming treatment were much more active than those under ambient temperature. Although increased activity often involves increased feeding time [20], it can also be associated with increased energy cost. Accordingly, we speculate that increased activity might explain the non-significant warming effect on tadpole growth in this study in the absence of predators.

In the presence of predator, however, warming increased tadpole growth in terms of body fresh weight. Specifically, between the last two observations tadpole survival remained constant, but the difference in fresh weight between the W−P+ and W+P+ treatments was greatly increased, such that a significant warming effect emerged. This is consistent with the two previous studies simultaneously and experimentally addressing the effect of predators and warming on tadpole growth. The study of Anderson et al. [5] shows that tadpole growth of *Hyla regilla* is significantly increased by warming in the presence of predators; Touchon and Warkentin [22] foiund that tadpole growth in Neotropical treefrog *Dendropsophus ebraccatus* was significantly improved only when predators were present. Differing from the experimental design of Anderson et al. [5], here we included the predator-free control to remove the potential confounding effects resulting from the warming effect on natural mortality and analyzed the warming effect between treatments with similar tadpole density. More importantly, unlike in many previous studies that used caged predators [22], in this study we allowed the predators to move freely around inside the water tank for assessing the impact of predation in a real world, which could produce substantially different outcomes from predator cues [43]. Thus, our results about warming effect on the prey-predator interaction are more likely to reflect what happens in nature.

This pronounced warming effect (in the presence of predators) on tadpole growth could be simply due to thinning and selection effect by predators. Over the course of our experiment, tadpole density decreased rapidly in the first three weeks in treatments with predators. Although food availability was non-limiting, the intraspecific interference and competition can be strong in the early experimental stages with high density of tadpoles. The release from the competition would promote tadpole growth in the later stages. Moreover, many tadpole predators, like the gape-limited diving beetles in our study, prefer to feed on small tadpoles because large ones are harder to handle and more likely to escape [25]. This would indirectly increase the average size of the remaining tadpoles. However, these two factors might not be effective in our case, because the tadpole survival was indistinguishable between the warming and ambient temperature treatments in the presence of predators. Instead, we postulate that warming might have increased the activity of beetles and thereby reduced the activity of tadpoles, which could have facilitated tadpole growth. Previous studies have shown that tadpoles often reduce activity level under predation risks for behavioral avoidance and that tadpole mortality rate increases with increasing activity level [44]–[46]. Indeed, in the W+P+ treatment tadpoles were more often observed perching on vegetation compared to those in the W−P+ treatment (Zhao J. Y Personal observation). The reduced activity did not necessarily reduce the foraging success for the tadpoles because of non-limiting food availability, but rather allowed more time of digesting and resting, which could facilitate tadpole growth. It is also possible that the warming intensified the chemical signal released by the beetles particularly when tadpoles were captured and consumed, as suggested by Richards and Bull [14]. This would further strengthen the cues of predation risks to increase tadpole growth.

### Tadpole survival

Tadpole survival did not increase with increasing body growth in the presence of predators. Actually, tadpole mortality occurred mostly during the first three weeks, while during this period the difference in fresh weight had not emerged. This indicates that increased growth rate did not serve as a trait to defend against predators in our case. It also suggests that frequent examinations of tadpole growth and survival are necessary to understanding the underlying mechanism of the warming effect on tadpole growth and survival. If increased growth and survival are both found in a single observation at the end of an experiment, it is very likely to attribute the higher survival to increased growth; this could lead to taking a correlative relationship for a cause-effect. Equally important is that many studies have used the whole length or body length to characterize tadpole growth; this could be misleading when warming induces a morphological response, as found in the present study.

As an increased growth contributed little to the tadpole survival even under potentially increased predation risks due to warming, it is plausible that tadpoles may adopt changed body form as an effective approach for defense. An increase in the whole length under warming condition was found to be associated with a constant fresh weight for the first three observations, suggesting that growth in the whole length is stimulated in the body building design of the surviving tadpoles. Moreover, results showed that the tail length was not reduced at a given body length in the warming treatment compared to under the ambient temperature in the presence of predators; whereas in the absence of predators, the tail length was smaller in the warming treatment than under ambient temperature at a given body length. These suggest that an increase in the whole length and tail length might have been crucial to tadpole survival under predation risks in the warming treatment. The enlarged tails may act as a lure that distracts predator's attack from the more vulnerable body parts [47] and tail ripping can allow tadpoles that are grabbed by predators to escape with only nonlethal injury [12], [48]. Enlarged tails also permit tadpoles to swim faster so as to escape from predators [12], [13], [49]. Both mechanisms might have been simultaneously acting to avoid predation in our study. In addition, the decreased activity level of the tadpoles might reduce the detection by predators and hence served as an adaptive strategy for survival under predation risks.

In summary, we found a significant increase in tadpole growth but not survival in the presence of predators. Although predation risk was potentially increased by the warming, the tadpoles defended themselves from predation principally by changing body form but not growth rate during the critical survival period. Our result suggests that the frequency of surveying population dynamics and associated parameters should be high enough to feature the prey-predator interactions and that the whole length is not a reliable indicator for tadpole growth. Provided that the beetle is the fiercest predator on the plateau frog and its predation success on the tadpoles is unchanged by warming, we suggest that the survival of this top predator species is not likely to benefit from the increased growth in a warming world.

## Supporting Information

Appendix S1 **The variation in daily mean temperature for warmed (red) and ambient (black) treatments during the experiment period.**
(DOCX)

Appendix S2 **Size and weight of tadpoles of** ***Rana kukunoris*** **used in the experiment.**
(DOCX)

Appendix S3 **Result of two-way ANOVAs showing the effects of warming and predator, and their interaction effect on tadpole survival (A), body fresh weight (B), whole length (C) and tail length (D) on each observation day.**
(DOCX)

Appendix S4 **The scaling relationship between body length and tail length, showing the slopes and intercepts for four treatments including warmed and predator present (W+P+), ambient and predator present (W−P+), warmed and predator absent (W+P−), and ambient and predator absent (W−P−).**
(DOCX)
