# Supplementary material for: Artificial Warming Facilitates Growth but Not Survival of Plateau Frog (Rana kukunoris) Tadpoles in Presence of Gape-Limited Predatory Beetles
Source: PLoS One. 2014 Jun 6;9(6):e98252. doi: 10.1371/journal.pone.0098252 (PMC4048183; doi:10.1371/journal.pone.0098252)
Supplement: Appendix S1 — The variation in daily mean temperature for warmed (red) and ambient (black) treatments during the experiment period. (DOCX) [file pone.0098252.s001.docx]

**Appendix S1** The variation in daily mean temperature for warmed (red) and ambient (black) treatments during the experiment period.
