# Supplementary material for: Artificial Warming Facilitates Growth but Not Survival of Plateau Frog (Rana kukunoris) Tadpoles in Presence of Gape-Limited Predatory Beetles
Source: PLoS One. 2014 Jun 6;9(6):e98252. doi: 10.1371/journal.pone.0098252 (PMC4048183; doi:10.1371/journal.pone.0098252)
Supplement: Appendix S2 — Size and weight of tadpoles of Rana kukunoris used in the experiment. (DOCX) [file pone.0098252.s002.docx]

**Table S1** Size and weight of tadpoles of *Rana kukunoris* used in the experiment.

|  | W-P- | W-P+ | W+P- | W+P+ |
| --- | --- | --- | --- | --- |
| Whole length(mm) | 9.265±0.035 | 9.205±0.025 | 9.245±0.027 | 9.257±0.035 |
| Body length(mm) | 4.079±0.026 | 4.092±0.022 | 4.089±0.021 | 4.088±0.023 |
| Tail length(mm) | 5.177±0.020 | 5.173±0.023 | 5.172±0.021 | 5.174±0.022 |
| Tail muscle depth(mm) | 0.769±0.005 | 0.771±0.004 | 0.770±0.006 | 0.770±0.005 |
| Body fresh weight (mg) | 0.996±0.000 | 1.007±0.000 | 0.996±0.000 | 1.00±0.000 |

Note: No significant difference in above variables was found among treatments.
