# Supplementary material for: Artificial Warming Facilitates Growth but Not Survival of Plateau Frog (Rana kukunoris) Tadpoles in Presence of Gape-Limited Predatory Beetles
Source: PLoS One. 2014 Jun 6;9(6):e98252. doi: 10.1371/journal.pone.0098252 (PMC4048183; doi:10.1371/journal.pone.0098252)
Supplement: Appendix S3 — Result of two-way ANOVAs showing the effects of warming and predator, and their interaction effect on tadpole survival (A), body fresh weight (B), whole length (C) and tail length (D) on each observation day. (DOCX) [file pone.0098252.s003.docx]

**Table S2** Result of two-way ANOVAs showing the effects of warming and predator, and their interaction effect on tadpole survival (A), body fresh weight (B), whole length (C) and tail length (D) on each observation day.

A Tadpole survival

| Date(day) |  | MS | DF | SS | F | P |
| --- | --- | --- | --- | --- | --- | --- |
| 0.5 | Warm | 26.45 | 1 | 26.45 | 1.57 | 0.228 |
|  | Predator | 530.45 | 1 | 530.45 | 31.48 | <.001 |
|  | Warm*predator | 48.05 | 1 | 48.05 | 2.85 | 0.111 |
|  | Error | 16.85 | 16 | 269.60 |  |  |
| 1 | Warm | 14.45 | 1 | 14.45 | 0.59 | 0.455 |
|  | Predator | 708.05 | 1 | 708.05 | 28.70 | <.001 |
|  | Warm*predator | 36.45 | 1 | 36.45 | 1.48 | 0.242 |
|  | Error | 24.66 | 16 | 394.8 |  |  |
| 2 | Warm | 4.05 | 1 | 4.05 | 0.15 | 0.706 |
|  | Predator | 858.05 | 1 | 858.05 | 31.17 | <.001 |
|  | Warm*predator | 18.05 | 1 | 18.05 | 0.66 | 0.430 |
|  | Error | 27.53 | 16 | 440.40 |  |  |
| 3 | Warm | 1.25 | 1 | 1.25 | 0.04 | 0.843 |
|  | Predator | 938.45 | 1 | 938.45 | 30.42 | <.001 |
|  | Warm*predator | 11.25 | 1 | 11.25 | 0.36 | 0.554 |
|  | Error | 30.85 | 16 | 493.60 |  |  |
| 7 | Warm | 0.00 | 1 | 0.00 | 0.00 | 1.000 |
|  | Predator | 3075.20 | 1 | 3075.20 | 70.09 | <.001 |
|  | Warm*predator | 5.00 | 1 | 5.00 | 0.11 | 0.740 |
|  | Error | 43.88 | 16 | 702.00 |  |  |
| 14 | Warm | 8.45 | 1 | 8.45 | 0.19 | 0.668 |
|  | Predator | 4176.05 | 1 | 4176.05 | 94.37 | <.001 |
|  | Warm*predator | 2.45 | 1 | 2.45 | 0.06 | 0.817 |
|  | Error | 44.30 | 16 | 708.00 |  |  |
| 21 | Warm | 151.25 | 1 | 151.25 | 2.77 | 0.116 |
|  | Predator | 5216.45 | 1 | 5216.45 | 95.54 | <.001 |
|  | Warm*predator | 54.45 | 1 | 54.45 | 1.00 | 0.333 |
|  | Error | 54.60 | 16 | 873.60 |  |  |
| 28 | Warm | 180.00 | 1 | 180.00 | 3.56 | 0.076 |
|  | Predator | 4620.80 | 1 | 4620.80 | 92.09 | <.001 |
|  | Warm*predator | 72.20 | 1 | 72.20 | 1.44 | 0.248 |
|  | Error | 50.20 | 16 | 802.80 |  |  |

B Body fresh weight

| Date(day) |  | MS | DF | SS | F | P |
| --- | --- | --- | --- | --- | --- | --- |
| 0.5 | Warm | 0.17 | 1 | 0.17 | 0.94 | 0.346 |
|  | Predator | 0.00 | 1 | 0.00 | 0.00 | 0.973 |
|  | Warm*predator | 0.13 | 1 | 0.13 | 0.71 | 0.413 |
|  | Error | 0.18 | 16 | 2.92 |  |  |
| 3 | Warm | 0.87 | 1 | 0.12 | 0.87 | 0.366 |
|  | Predator | 0.08 | 1 | 0.01 | 0.08 | 0.788 |
|  | Warm*predator | 0.37 | 1 | 0.05 | 0.37 | 0.553 |
|  | Error | 0.14 | 16 | 2.19 |  |  |
| 7 | Warm | 0.04 | 1 | 0.04 | 0.47 | 0.501 |
|  | Predator | 0.01 | 1 | 0.01 | 0.09 | 0.768 |
|  | Warm*predator | 0.00 | 1 | 0.00 | 0.06 | 0.811 |
|  | Error | 0.07 | 16 | 1.20 |  |  |
| 14 | Warm | 0.01 | 1 | 0.01 | 0.14 | 0.717 |
|  | Predator | 0.07 | 1 | 0.07 | 1.44 | 0.247 |
|  | Warm*predator | 0.00 | 1 | 0.00 | 0.04 | 0.853 |
|  | Error | 0.05 | 16 | 0.74 |  |  |
| 21 | Warm | 0.01 | 1 | 0.01 | 0.50 | 0.490 |
|  | Predator | 0.42 | 1 | 0.42 | 25.71 | 0.000 |
|  | Warm*predator | 0.09 | 1 | 0.09 | 5.25 | 0.036 |
|  | Error | 0.02 | 16 | 0.26 |  |  |
| 28 | Warm | 0.03 | 1 | 0.03 | 2.80 | 0.114 |
|  | Predator | 0.44 | 1 | 0.44 | 38.28 | <.001 |
|  | Warm*predator | 0.12 | 1 | 0.12 | 10.82 | 0.005 |
|  | Error | 0.01 | 16 | 0.18 |  |  |

C Whole length

| Date(day) |  | MS | DF | SS | F | P |
| --- | --- | --- | --- | --- | --- | --- |
| 0.5 | Warm | 0.02 | 1 | 0.02 | 0.13 | 0.721 |
|  | Predator | 0.00 | 1 | 0.00 | 0.03 | 0.873 |
|  | Warm*predator | 0.03 | 1 | 0.03 | 0.16 | 0.692 |
|  | Error | 0.17 | 16 | 2.69 |  |  |
| 3 | Warm | 0.60 | 1 | 0.60 | 3.15 | 0.095 |
|  | Predator | 4.15 | 1 | 4.15 | 21.84 | 0.000 |
|  | Warm*predator | 1.45 | 1 | 1.45 | 7.62 | 0.014 |
|  | Error | 0.19 | 16 | 3.04 |  |  |
| 7 | Warm | 2.47 | 1 | 2.47 | 9.86 | 0.006 |
|  | Predator | 9.22 | 1 | 9.22 | 36.87 | <.001 |
|  | Warm*predator | 0.06 | 1 | 0.06 | 0.25 | 0.621 |
|  | Error | 0.25 | 16 | 4.00 |  |  |
| 14 | Warm | 11.91 | 1 | 11.91 | 34.92 | <.001 |
|  | Predator | 29.92 | 1 | 29.92 | 87.72 | <.001 |
|  | Warm*predator | 4.38 | 1 | 4.38 | 12.84 | 0.003 |
|  | Error | 0.34 | 16 | 5.46 |  |  |
| 21 | Warm | 25.58 | 1 | 25.58 | 15.39 | 0.001 |
|  | Predator | 45.78 | 1 | 45.78 | 27.54 | <.001 |
|  | Warm*predator | 14.74 | 1 | 14.74 | 8.87 | 0.009 |
|  | Error | 1.66 | 16 | 26.60 |  |  |
| 28 | Warm | 16.18 | 1 | 16.18 | 8.68 | 0.010 |
|  | Predator | 101.96 | 1 | 101.96 | 54.72 | <.001 |
|  | Warm*predator | 25.19 | 1 | 25.19 | 13.52 | 0.002 |
|  | Error | 1.86 | 16 | 29.82 |  |  |

D Tail length

| Date(day) |  | MS | DF | SS | F | P |
| --- | --- | --- | --- | --- | --- | --- |
| 0.5 | Warm | 0.01 | 1 | 0.01 | 0.62 | 0.441 |
|  | Predator | 0.00 | 1 | 0.00 | 0.04 | 0.843 |
|  | Warm*predator | 0.00 | 1 | 0.00 | 0.10 | 0.761 |
|  | Error | 0.02 | 16 | 0.34 |  |  |
| 3 | Warm | 0.17 | 1 | 0.17 | 2.38 | 0.142 |
|  | Predator | 1.16 | 1 | 1.16 | 16.55 | 0.001 |
|  | Warm*predator | 0.76 | 1 | 0.76 | 10.84 | 0.005 |
|  | Error | 0.07 | 16 | 1.12 |  |  |
| 7 | Warm | 0.95 | 1 | 0.95 | 8.58 | 0.010 |
|  | Predator | 2.31 | 1 | 2.31 | 20.92 | 0.000 |
|  | Warm*predator | 0.08 | 1 | 0.08 | 0.70 | 0.416 |
|  | Error | 0.11 | 16 | 1.77 |  |  |
| 14 | Warm | 3.13 | 1 | 3.13 | 16.85 | 0.001 |
|  | Predator | 8.45 | 1 | 8.45 | 45.45 | <.001 |
|  | Warm*predator | 1.45 | 1 | 1.45 | 7.78 | 0.013 |
|  | Error | 0.19 | 16 | 2.98 |  |  |
| 21 | Warm | 8.79 | 1 | 8.79 | 16.62 | 0.001 |
|  | Predator | 14.48 | 1 | 14.48 | 27.36 | <.001 |
|  | Warm*predator | 5.15 | 1 | 5.15 | 9.72 | 0.007 |
|  | Error | 0.53 | 16 | 8.47 |  |  |
| 28 | Warm | 6.22 | 1 | 6.22 | 7.43 | 0.015 |
|  | Predator | 37.85 | 1 | 37.85 | 45.24 | <.001 |
|  | Warm*predator | 9.22 | 1 | 9.22 | 11.02 | 0.004 |
|  | Error | 0.84 | 16 | 13.38 |  |  |
