# Supplementary material for: Artificial Warming Facilitates Growth but Not Survival of Plateau Frog (Rana kukunoris) Tadpoles in Presence of Gape-Limited Predatory Beetles
Source: PLoS One. 2014 Jun 6;9(6):e98252. doi: 10.1371/journal.pone.0098252 (PMC4048183; doi:10.1371/journal.pone.0098252)
Supplement: Appendix S4 — The scaling relationship between body length and tail length, showing the slopes and intercepts for four treatments including warmed and predator present (W+P+), ambient and predator present (W−P+), warmed and predator absent (W+P−), and ambient and predator absent (W−P−). (DOCX) [file pone.0098252.s004.docx]

**Table S3** The scaling relationship between body length and tail length, showing the slopes and intercepts for four treatments including warmed and predator present (W+P+), ambient and predator present (W-P+), warmed and predator absent (W+P-), and ambient and predator absent (W-P-). The data were collected in three days after the beginning of the experiment, when predator-induced defensive traits were present.

| *y* | *x* | Group | N | R^2^ | P | Slope | LowCI | UppCI | Interc |
| --- | --- | --- | --- | --- | --- | --- | --- | --- | --- |
| Tail length | Body length | W-P- | 1137 | 0.770 | <.001 | 0.912 | 0.897 | 0.928 | 0.228 |
|  |  | W+P- | 1214 | 0.862 | <.001 | 0.912 | 0.897 | 0.928 | 0.224 |
|  |  | W-P+ | 687 | 0.736 | <.001 | 1.056 | 1.034 | 1.077 | 0.096 |
|  |  | W+P+ | 666 | 0.831 | <.001 | 1.056 | 1.034 | 1.077 | 0.096 |
